# Microphysiological gut-on-chip enables extended *in vitro* development of *Cryptosporidium hominis*

**DOI:** 10.3389/fcimb.2025.1564806

**Published:** 2025-04-24

**Authors:** Samantha Gunasekera, Benjamin Thierry, Brendon King, Paul Monis, Jillian M. Carr, Abha Chopra, Mark Watson, Mark O’Dea, Edward Cheah, Ramesh Ram, Peta L. Clode, Nawal Hijjawi, Una Ryan

**Affiliations:** ^1^ Harry Butler Institute, College of Environmental and Life Sciences, Murdoch University, Murdoch, WA, Australia; ^2^ Future Industries Institute, University of South Australia, Adelaide, SA, Australia; ^3^ Australian Water Quality Centre, South Australian Water Corporation, Adelaide, SA, Australia; ^4^ Flinders Health and Medical Research Institute, College of Medicine and Public Health, Flinders University, Bedford Park, SA, Australia; ^5^ Immunology and Infectious Diseases, Murdoch University, Murdoch, WA, Australia; ^6^ Centre for Microscopy, Characterisation, and Analysis and School of Biological Sciences, The University of Western Australia, Crawley, WA, Australia; ^7^ Department of Medical Laboratory Sciences, Faculty of Applied Health Sciences, The Hashemite University, Zarqa, Jordan

**Keywords:** *Cryptosporidium hominis*, gut-on-chip, HCT-8 cells, fluid shear stress, *in vitro*

## Abstract

**Introduction:**

*Cryptosporidium hominis* is the dominant *Cryptosporidium* species infecting humans, but most advances in developing robust *in vitro* culturing platforms for *Cryptosporidium* have utilised *C. parvum*. Consequently, there is relatively little available information specific to the biology and life cycle of *C. hominis*. The present study utilised a pumpless and tubeless gut-on-chip to generate a physiologically relevant *in vitro* environment by applying a constant fluid shear stress of 0.02 dyn cm^-2^ to HCT-8 cells.

**Methods:**

Gut-on-chips were fabricated using standard soft lithography. *C. hominis* oocysts isolated from human pathology samples were used to infect the human ileocecal colorectal adenocarcinoma (HCT-8) cell line under a constant fluid shear stress of 0.02 dyn cm^-2^. Parasite growth was assessed using a *C. hominis*-specific quantitative PCR, a *Cryptosporidium* genus-specific immunofluorescence assay, and scanning electron microscopy. Differences in the HCT-8 transcriptome with and without fluid shear stress, and the host-parasite interaction, were both assessed using bulk transcriptomics.

**Results:**

Transcriptomic analysis of the HCT-8 cell line cultured within the gut-on-chip demonstrated a metabolic shift towards oxidative phosphorylation when compared to the same cell line cultured under static conditions. Extended *C. hominis* (subtype IdA15G1) cultures were sustained for up to 10 days within the gut-on-chip as shown by a *C. hominis*-specific qPCR and a *Cryptosporidium* genus-specific immunofluorescence assay, which demonstrated ~30-fold amplification in the gut-on-chip over the duration of the experiment. Scanning electron microscopy of infected monolayers identified trophozoites, meronts, merozoites, macrogamonts, microgamonts, and possible gamont-like stages at 48 h post-infection. The potential role of gamonts in the *Cryptosporidium* life cycle remains unclear and warrants further investigation. Transcriptomes of HCT-8 cells infected with *C hominis* revealed upregulation of biological processes associated with cell cycle regulation and cell signalling in *C. hominis*-infected cells under fluid shear stress compared to static culture.

**Conclusions:**

These data demonstrate that bioengineered gut-on-chip models support extended *C. hominis* growth and can be used to interrogate responses of host cells to infection. Owing to its relative simplicity, the pumpless and tubeless gut-on-chip can be accessible to most laboratories with established HCT-8 infection models for *Cryptosporidium* culture.

## 1 Introduction

Parasites of the *Cryptosporidium* genus are one of the leading causes of severe diarrhoea-associated disease and death in young children in sub-Saharan Africa and South Asia (Buchwald et al., 2023; Nasrin et al., 2021). Together, *C. hominis* and *C. parvum* are responsible for over 95% of human associated outbreaks of cryptosporidiosis globally (Yang et al., 2021). Despite *C. hominis* accounting for the largest share of human cryptosporidiosis cases in most parts of the world (Yang et al., 2021), most existing knowledge of the biology of *Cryptosporidium* infection in humans is based on investigating *C. parvum* (English et al., 2022; Tandel et al., 2019; Guérin et al., 2021). The bias in studies on *Cryptosporidium* development towards *C. parvum* is mainly a result of the relative ease of acquiring *C. parvum* oocysts, due to its wider host range and consequent ability to propagate these oocysts in other animals (Feng et al., 2018). The lack of information on *C. hominis* has been exacerbated by its relatively recent classification as a separate species (Morgan-Ryan et al., 2002), with previous research referring to “*C. parvum* genotype H” or “genotype 1”. Additionally, while there has been a wealth of advancements in *in vitro* culture systems for *C. parvum* (Morada et al., 2016; DeCicco RePass et al., 2017; Baydoun et al., 2017; Heo et al., 2018; Wilke et al., 2019; Nikolaev et al., 2020; Lamisere et al., 2022), understanding of *C. hominis* has fallen behind, with substantially fewer investigations of the *in vitro* development of this species (Hijjawi et al., 2010; Greigert et al., 2024; Hashim et al., 2004, 2006; Su et al., 2019).

Research into the biology of *C. hominis* infection has largely relied on the *in vivo* gnotobiotic piglet model (Lee et al., 2017, 2018, 2019; Widmer et al., 2000), the *in vivo* immunosuppressed gerbil model (Baishanbo et al., 2005), or investigations limited to the early stages of *C. hominis* infection using the *in vitro* HCT-8 model (Hashim et al., 2004, 2006; Su et al., 2019). More recently, a human ileal stem cell-derived air-liquid interface culture system has been described to support complete development of *C. hominis* (Greigert et al., 2024). While most available information suggests that *C. hominis* invades enterocytes using a mechanism distinct from *C. parvum* (Su et al., 2019; Hashim et al., 2004, 2006), most other aspects of the *in vitro* development of *C. hominis* are assumed to be similar to that of *C. parvum* (Hijjawi et al., 2001; Bhat et al., 2007; Greigert et al., 2024), or remain incompletely understood. In addition, very little subtype-specific information on the *in vitro* development of *Cryptosporidium* is available (Gunasekera et al., 2023).

This study describes the extended *in vitro* development of *C. hominis* using pumpless and tubeless microfluidics. The use of microfluidics enables the application of fluid flow, and the resulting fluid shear stress to *in vitro* cell cultures provides a more physiologically relevant representation of the *in vivo* environment (Kim et al., 2012; Kim and Ingber, 2013). The control of fluid flow in conventional microfluidic devices using external pumps and tubing, increases the risk of air bubble formation and fluid leakages; the former of which is destructive to the cell monolayer, and the latter of which creates unacceptable biological risk. The use of pumpless and tubeless microfluidics bypasses both obstacles (Sung et al., 2019). The application of fluid shear stress to cell cultures has gained popularity as a vehicle to study other enteric pathogens (Grassart et al., 2019; Chi et al., 2015; Tovaglieri et al., 2019; Sunuwar et al., 2020; Villenave et al., 2017), including *C. parvum* (Nikolaev et al., 2020; Gunasekera et al., 2024). The experiments described herein utilised *C. hominis* (subtype IdA15G1) oocysts; a relevant subtype involved in recent swimming pool-associated outbreaks in Western Australia (Braima et al., 2021). The gut-on-chip described in the present study will assist future research in further understanding the progression of *C. hominis* infection and help to close the gap between our knowledge and understanding of *C. hominis* in respect to *C. parvum*.

## 2 Methods

### 2.1 Fabrication of microfluidic devices

**Figure 1:**
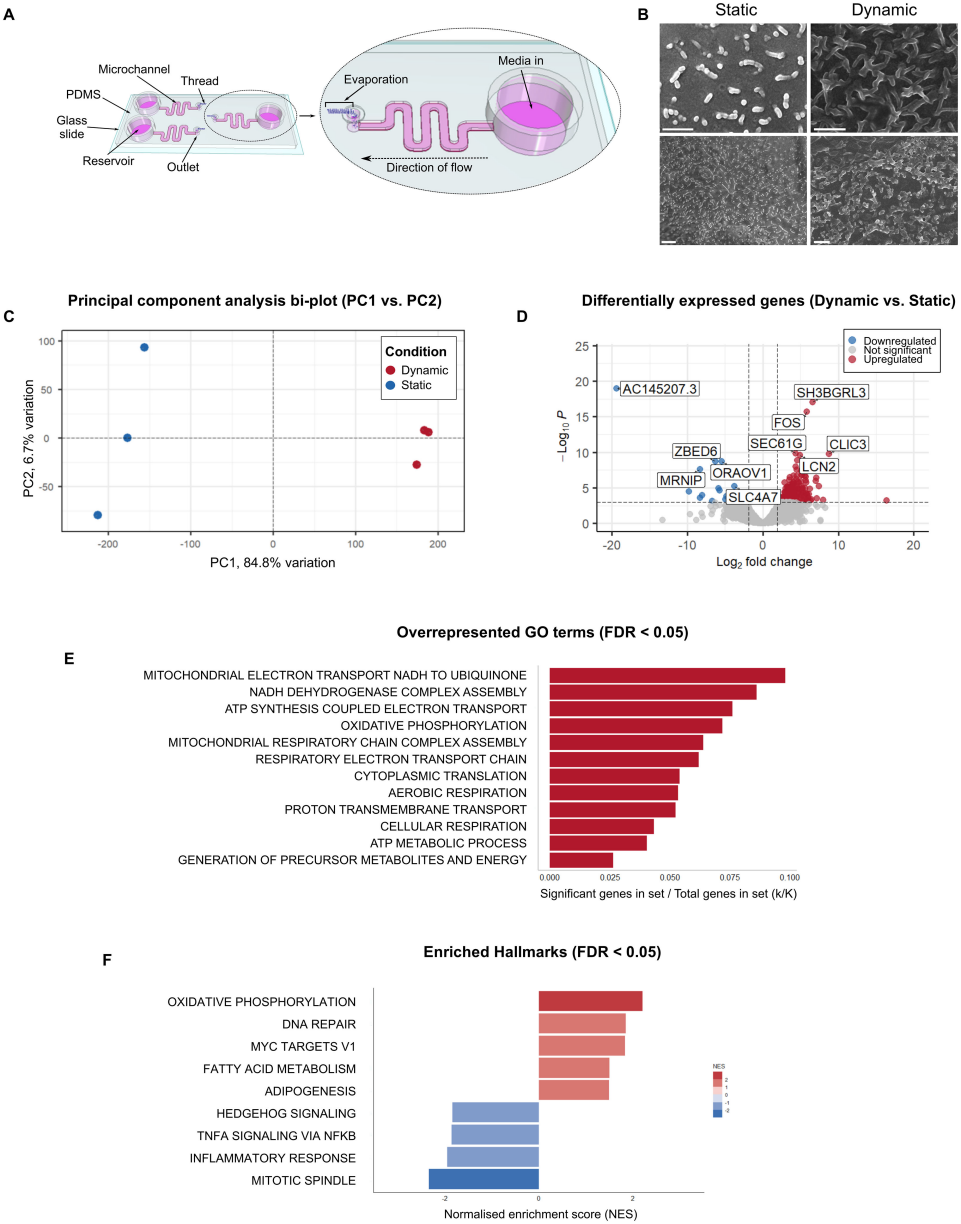
HCT-8 cells cultured within microfluidic devices have a distinct morphological and transcriptomic profile. **(A)** Schematic diagram of the gut-on-chip (adapted from Gunasekera et al., 2024) demonstrating the location of each microchannel, reservoir, outlet and thread, and total volume of each microchannel and reservoir (not drawn to scale). **(B)** Scanning electron micrographs of HCT-8 monolayers cultured on either borosilicate glass coverslips (left) or within microfluidic devices (right) until 90% confluent, fixed with glutaraldehyde, dehydrated using graded ethanol and hexamethyldisilazane, and coated with 5 nm platinum. Scale bars = 1 µm. **(C)** The principal component analysis bi-plot showed that PC1 and PC2 account for 84.8% and 6.7% variation across all samples respectively. **(D)** Differential expression analysis indicated the presence of 219 statistically significantly differentially expressed genes between the two treatment groups. The five top up-regulated genes (*SH3BGRL3*, *FOS*, *SEC61G*, *CLIC3*, *LCN2*) and the five top-downregulated genes (AC145207.3, *ZBED6*, *ORAOV1*, *MRNIP*, *SLC4A7*) are annotated on the plot. Functional annotation of these genes is provided in **Supplementary Table 2**. **(E)** Overrepresented GO terms among the 177 significantly upregulated genes in HCT-8 cells cultured under fluid shear stress (0.02 dyn cm-2), normalised for the size of each gene set (FDR ≤ 0.05), **(F)** Gene set enrichment analysis using the Hallmark gene sets against our gene list containing the entire dataset, irrespective of statistical significance, ranked from highest to lowest log_2_-fold change. This analysis revealed nine enriched Hallmarks in the HCT-8 transcriptome when cultured under fluid shear stress. Positive normalised enrichment scores shown in red represent gene sets that were enriched at the top of the gene list (i.e. upregulated), and negative normalised enrichment scores shown in blue represent gene sets enriched at the bottom of the gene list (i.e. downregulated).

The gut-on-chips utilised in this study were fabricated using standard soft lithography, following the methods described in detail previously (Delon et al., 2020). Briefly, a master mold was created by spin-coating SU-8 photoresist onto a silicon wafer, with a mask writer used to generate the microstructures that form the pattern of each microchannel. The SU-8 photoresist was then subject to thermal hardening to form the final master mold. A 10 mm thick PDMS layer with a curing agent (10:1) was cast onto the master mold and incubated at 65°C for 2 h. The PDMS layer was then peeled from the master mold, cut to size and an inlet reservoir and outlet were created using an 8 mm and 1.5 mm hole punch respectively. The microchannel surface of the PDMS layer was then treated with air plasma for three minutes, pressed onto the cover glass, then incubated at 65°C for 10 minutes to bond the glass and PDMS layers. The resulting gut-on-chips comprised of a layer of polydimethylsiloxane (PDMS) bound to a glass coverslip. The PDMS layer contained three independent microchannels (L: 36 mm, W: 1 mm, H: 155 µm, volume: 5.25 µL), each connected to a reservoir (diameter: 8 mm, volume: 200 µL) and an outlet (diameter: 1.5 mm). The glass coverslip formed the basal surface of each microchannel (**Figure 1A**). In cases where the gut-on-chips were required for scanning electron microscopy (SEM), a thicker microscope slide was used in place of a coverslip to facilitate sample handling and processing.

### 2.2 HCT-8 cell culture

All experiments utilised a human ileocecal colorectal adenocarcinoma cell line (HCT-8, ATCC CCL-244). The cells were routinely cultured in 75 cm^2^ flasks with a complete growth medium comprising RPMI-1640 (Sigma) supplemented with 10% (v/v) foetal bovine serum (FBS, Bovogen), 1% (v/v) penicillin-streptomycin (Sigma), 2 mM L-glutamine (Sigma), and 15 mM HEPES (Sigma), pH 7.2. The cell line was confirmed negative for Mycoplasma contamination prior to experiments as described previously (Young et al., 2010), and all experiments were performed between 60 and 100 cell doublings. HCT-8 cells grown under fluid shear stress conditions were cultured within gut-on-chips, while static comparison experiments without fluid shear stress were performed using 24-well plates. Cell suspensions were prepared by trypsinisation of the cell monolayer for six minutes at 37°C. The trypsin-EDTA (Sigma) was inactivated using two volumes of complete growth medium.

Prior to seeding the gut-on-chips with HCT-8 cells, the microchannels were rinsed with 70% (v/v) ethanol (Chem-Supply) and PBS three times each. The glass surface of each microchannel was then coated with a 1% (v/v) Matrigel (Corning) solution by dispensing the solution slowly from the outlet at 4°C, followed by immediate incubation at 37°C for 60 min. 2 x 10^5^ HCT-8 cells were injected into each microchannel and grown without fluid flow for 16 h in order to promote cell adherence to the Matrigel-coated surface. The inlet reservoir was topped up with complete growth medium to prevent the cells from drying out. Fluid flow was initiated by insertion of a hydrophilic, spunlace thread (70% rayon, 30% polyester, Ebos Healthcare Australia) into each outlet using sterile, fine-tipped forceps. Each thread with a width of 1 mm was hand-cut to a length of 10 mm and autoclaved prior to use. HCT-8 cell monolayers were then grown to confluence under a constant fluid shear stress (0.02 dyn cm^-2^). For static comparison experiments, HCT-8 cells were seeded into 24-well plates at a density of 5 x 10^4^ cells per well and grown to confluence with complete growth medium.

### 2.3 Collection, purification, and genotyping of *C. hominis* oocysts

The *C. hominis* oocysts were acquired from human clinical cryptosporidiosis samples (*n* = 5) from a local pathology laboratory (Perth, Australia). The oocysts were isolated from faecal material using a Ficoll-density centrifugation protocol outlined previously (Morgan et al., 1995), with modifications as described previously (Gunasekera et al., 2023). Following purification, the oocyst isolates were stored at 4°C in PBS supplemented with 1% (v/v) penicillin-streptomycin and 1% (v/v) amphotericin B prior to experiments. All oocyst isolates were used for experiments within 16 weeks of purification.

Samples were confirmed as *C. hominis* using a previously described quantitative PCR (qPCR) assay targeting a genus-specific locus and a *C. hominis-*specific probe (Yang et al., 2013), followed by subtyping at the 60-kilodalton glycoprotein (*gp60*) locus using a nested PCR primer set described previously (Peng et al., 2001). Briefly, template DNA was extracted from an aliquot of 1 x 10^5^ oocysts per sample, which was freeze-thawed five times via alternating incubation periods in liquid nitrogen for one minute and on a 100°C heating block for two minutes. The DNA from each lysate (2 µL lysate diluted 1:100) was amplified using the CFX96 Touch Real-Time PCR Detection System (Bio-Rad) in a 10 µL reaction comprising 1X GoTaq Probe qPCR Master Mix (Promega), 0.6 µM EVF1 forward primer (5′-GAA CTG TAC AGA TGC TTG GGA GAA T), 0.6 µM EVR1 reverse primer (5′-CCTT CGT TAG TTG AAT CCT CTT TCC A) and 0.25 µM minor groove-binding (MGB) probe (5’-FAM – TTG GAG CTC ATA TCA G 3’ – MGBNFQ), which was designed to be specific at the species level to *C. hominis* (Yang et al., 2013). The following cycling conditions were used: 95°C for 2 minutes, then 45 cycles of 95°C for 30 seconds and 60°C for 60 seconds. The data were analysed using the CFX Manager (Bio-Rad) software. Reagent concentrations for *gp60* subtyping were as described previously (Gunasekera et al., 2023), and amplification was carried out using a C1000 Thermal Cycler (BioRad) under cycling conditions described previously (Strong et al., 2000). The secondary PCR products were subject to agarose gel electrophoresis using a 1% gel followed by Sanger sequencing. The *gp60* subtype was verified using sequence alignments against an in-house database with the Clustal W alignment tool (Larkin et al., 2007).

### 2.4 Excystation pre-treatment of *C. hominis* oocysts, sporozoite purification and infection of HCT-8 cells

Once confirmed as *C. hominis*, oocysts were quantified by manual counting with a haemocytometer, then incubated in a 0.25% (v/v) sodium hypochlorite solution for 30 minutes at 4°C. Excystation pre-treatment of oocysts was performed as described previously (King et al., 2015). The excystation pre-treated oocysts were resuspended at a concentration of 5 x 10^3^ oocysts and a volume of 20 µL per microchannel. The infection medium comprised RPMI-1640 supplemented with 2 mM L-glutamine, 5.6 mM glucose, 0.02% (w/v) bovine bile, 15 mM HEPES, 0.6 µM folic acid, 7.3 µM 4-aminobenzoic acid, 2.1 µM calcium pantothenate, 50 µM L-ascorbic acid, 2.5 µg mL^-1^ amphotericin B, 1% (v/v) penicillin-streptomycin (all from Sigma) and 1% (v/v) FBS, pH 7.2.

For experiments requiring purified sporozoites, the excystation-pretreated oocysts were resuspended in 1 mL infection medium and incubated at 37°C for 45 min. The oocyst shells and unexcysted oocysts were filtered out using a 2 µm syringe filter (Whatman). The filter was then rinsed with 1 mL infection medium. All filtrate was then centrifuged at 3,200 x *g* for 20 minutes and resuspended in 60 µL infection medium, corresponding to 4 x 10^5^ sporozoites per microchannel. The thread was removed from each outlet prior to application of *C. hominis* to the HCT-8 monolayer, then suspensions of either excystation pre-treated oocysts or purified sporozoites were injected into each microchannel directly from the outlet in a volume of 20 µL. The infection medium in each reservoir was topped up, then each gut-on-chip was incubated without the thread for four hours at 37°C and 5% CO_2_ in a humidified incubator. The fluid flow was re-initiated by inserting a fresh hydrophilic thread into the outlet of each microchannel. For static comparison experiments, conventional *Cryptosporidium* co-cultures with HCT-8 cells were prepared using standard protocols utilised in routine *Cryptosporidium* infectivity testing (King et al., 2011, 2015). Briefly, excystation pre-treated oocysts were applied directly to confluent HCT-8 monolayers in 24-well plates, then each plate was centrifuged at 410 x *g* for five minutes before incubation at 37°C and 5% CO_2_ in a humidified incubator. The cell monolayers were gently rinsed with PBS after four hours to remove any oocyst shells from the monolayer. The infection medium was refreshed at 24 h intervals for the duration of each experiment, and all spent media was reserved in 1.5 mL tubes for further analysis with quantitative PCR.

### 2.5 Parasite enumeration using qPCR and immunofluorescence assays (Sporo-Glo Ab)

For experiments designed to quantify *C. hominis* infection over time, a total of 16 microchannels were seeded with HCT-8 cells, and once the monolayers were grown to confluence under constant fluid shear stress conditions (0.02 dyn cm^-2^), 15 of these microchannels were infected with excystation pre-treated oocysts as described above. The remaining uninfected microchannel was designated as the negative control for immunostaining and the no-template control for qPCR assays. For static comparison experiments, 16 wells were seeded with HCT-8 cells and 15 of these were infected with excystation pre-treated oocysts. All relevant steps for downstream experiments remained the same between both culture systems.

To determine whether HCT-8 cell monolayers contained actively dividing *Cryptosporidium* life cycle stages, three microchannels were set aside every 48 h for the duration of each experiment for immunofluorescence assays. Such experiments utilised a commercially available fluorescein-labelled rat anti-*Cryptosporidium* polyclonal antibody (1X Sporo-Glo, Waterborne Inc.), that binds to all intracellular dividing *Cryptosporidium* life cycle stages, using a previously described protocol (King et al., 2011). Immediately prior to sample preparation for immunofluorescence assays, the thread was removed from each outlet, immersed in 20 µL infection medium and stored at 4°C for downstream qPCR. Each microfluidic device was imaged using an Eclipse TS100 inverted fluorescence microscope (Nikon). Post-processing of all immunofluorescence images was performed using Adobe Photoshop CS6 v13.0 using a green levels adjustment layer with the following settings: shadow input 7, midtone input 1.8, highlight input 70.

To measure parasite load over time, a qPCR using the previously described EVF1 and EVR1 primers and *C. hominis*-specific MGB probe was utilised (Yang et al., 2013), with the reagent concentrations and cycling conditions described above. Following the immunofluorescence assays, each monolayer was treated with 0.25% (v/v) trypsin-EDTA at 37°C for a minimum of 30 minutes. The trypsin-EDTA was inactivated with one volume of infection medium, and each cell suspension was transferred to 1.5 mL tubes. All tubes from each sample (thread, harvested monolayers, residual infection medium) were centrifuged at 20,000 x *g* for 10 minutes, and the supernatant was removed from each tube leaving ~20 µL remaining. Each sample was then lysed by freeze-thawing five times using alternating one minute liquid nitrogen immersions with two-minute 100°C heating block incubations. The lysates were then stored at -20°C until ready for qPCR. DNA was amplified using the CFX96 Touch Real-Time PCR Detection System (Bio-Rad) using 2 µL lysate diluted 1:100 and a standard curve was created using serially diluted gBlocks dsDNA gene fragments (Integrated DNA Technologies) that were designed to be homologous to the region of interest.

### 2.6 Scanning electron microscopy

A SEM-based approach was employed to identify the presence of specific *C. hominis* life cycle stages. Gut-on-chips destined for SEM were fabricated using a microscope glass slide to facilitate PDMS layer removal. Confluent HCT-8 monolayers were infected with 1 x 10^5^ excystation pre-treated oocysts per microchannel, then at 48 h post-infection, the monolayers were fixed overnight at 4°C in 2.5% (v/v) glutaraldehyde in PBS with pH adjusted to 7.4. The following morning, the fixative was thoroughly rinsed off with three PBS washes. With the gut-on-chip immersed in PBS, the PDMS layer was carefully removed from the glass slide with a scalpel blade. Each sample was then subjected to graded ethanol dehydration and subsequent chemical dehydration using hexamethyldisilazane (Sigma). The sample was left to dry overnight in the fume hood, then sputter coated with 5 nm platinum for high resolution imaging. Uninfected HCT-8 monolayers cultured in microfluidic devices and on 10 mm borosilicate glass coverslips were also processed for SEM as described above. All data were acquired using a 55VP Supra field emission SEM (Zeiss), using the in-lens secondary electron detector and an accelerating voltage of 5 kV.

### 2.7 Detection of new oocysts produced *in vitro*

For experiments designed to determine whether new oocysts were being produced *in vitro*, HCT-8 monolayers were infected with purified *C. hominis* sporozoites instead of excystation pre-treated oocysts. Six microchannels were seeded with HCT-8 cells in total, and three of these were infected with 4 x 10^5^ purified sporozoites. All infection media inside each microchannel and each reservoir were collected in 1.5 mL tubes at 24 h intervals for seven days in total. Each tube was centrifuged at 8,000 x *g* for three minutes, then pellets were subject to two PBS washes and resuspended in 50 µL. Each sample was then prepared for immunostaining using a commercially available kit (EasyStain™, BioPoint) using the supplier’s instructions. The EasyStain™ antibody is conjugated to fluorescein isothiocyanate and binds specifically to *Cryptosporidium* oocyst wall proteins. Each sample was imaged using an Eclipse 80i Fluorescence Microscope (Nikon).

To determine whether potential oocysts produced *in vitro* could establish infection in a new HCT-8 cell culture, the medium from one microchannel was set aside at 72 h post-infection and applied to a fresh HCT-8 monolayer. The new HCT-8 monolayer was then prepared for immunostaining using 1X Sporo-Glo as described above to determine presence of *C. hominis* intracellular dividing stages. The sample was then imaged using an Eclipse TS100 inverted fluorescence microscope (Nikon).

### 2.8 Sample preparation for bulk RNA-seq

A transcriptomics approach was used to investigate the biological basis for enhanced *in vitro* development under fluid shear stress conditions. Six microfluidic devices and three plate wells were seeded with HCT-8 cells and grown to confluence. Three microfluidic devices were infected with 1 x 10^5^ excystation pre-treated *C. hominis* oocysts per microchannel as described above while the remaining HCT-8 cultures were left uninfected. At 48 h post-infection, all cell monolayers were rinsed twice with PBS and subject to a six-minute incubation with 0.25% (w/v) trypsin-EDTA at 37°C. The trypsin-EDTA was inactivated with two volumes of complete growth medium and then the cell suspensions from three microchannels were pooled together in falcon tubes to form three samples per condition. Each tube was centrifuged for ten minutes at 500 x *g*, and all supernatant was discarded. The pellet was then resuspended in 1 mL RNA*later* (Thermo Fisher) and each sample was incubated overnight at 4°C. The next morning, the RNA*later* was diluted with three volumes of RPMI-1640 and each tube was centrifuged at 1,800 x *g* for ten minutes. All supernatant was removed and discarded, then the RNeasy kit (Qiagen) was used for RNA extraction according to the manufacturer’s instructions. The RNA was stored at -80°C until ready for further processing.

The quality of each sample was assessed using the TapeStation system (Agilent), with an RNA integrity score of >7.0 deemed suitable for further processing. Ribosomal RNA depletion was undertaken using the NEBNext rRNA depletion kit V2 (New England Biolabs). cDNA conversion was performed using an adapted Smart-seq assay (Picelli et al., 2014), targeting all RNA transcripts using biotinylated oligo dT and random nonamer primers:/5Biosg/AAGCAGTGGTATCAACGCAGACATTTAGG (30T*V*(N)) and/5Biosg/AAGCAGTGGTATCAACGCAGACATTTAGGNNNNNNNNN respectively. Briefly, the first cDNA strand was synthesised with untemplated C nucleotides added to the 3’ end, creating a poly(C) overhang. The poly(C) overhang is only added to full length transcripts. A template switching oligo (TSO) (AAGCAGTGGTATCAACGCAGAGTACATrGrG+G) was hybridised to the poly(C) overhang and used to synthesise the second strand. The full-length transcripts were then amplified, purified and quantified using Promega Quantus Fluorometer (Promega Inc). Libraries were then prepared from the double stranded cDNA using NEBNext^®^ Ultra™ II FS DNA Library Prep Kit for Illumina (New England Biolabs) and sequenced on a NovaSeq 6000 using 150 bp PE Chemistry (Illumina Inc). Sequencing data were uploaded to the NCBI SRA database (BioProject accession number PRJNA1051824).

### 2.9 Transcriptomics data analysis

To compare the transcriptomes of HCT-8 cells cultured within the microfluidic device with HCT-8 cells cultured in 24-well plates, firstly the sequencing data was assessed for quality using FastQC (v0.11.9). Quantification of transcript abundance was undertaken using kallisto (v0.46.2) quant function using the human GRCh38.p12 (December 2013) reference genome (Lander et al., 2001), with the GRCh38 Ensembl release 93 (2018) annotation gtf file (Martin et al., 2022), under default settings, with the optional bias and bootstrap arguments used (Bray et al., 2016). The output from kallisto was then converted to gene-level counts using txImport (v1.22.0). The data were normalised using RUVSeq (v1.28.0) using the negative control genes listed in **Supplementary Table 1** (Risso et al., 2014). Low abundance transcripts were removed using RUVSeq to only retain data with > 5 counts in at least two samples, and any batch effects present were corrected using limma (v3.50.3) *removeBatchEffect* function (Ritchie et al., 2015). The log_2_ normalised gene counts were used for principal component analysis (Blighe and Lun, 2021), and the statistical significance of the results of principal component analysis was subsequently assessed (Camargo, 2022). A likelihood ratio test (LRT) using DESeq2 (v1.34.0) with the false discovery rate assigned as 0.05, was undertaken to identify differentially expressed genes between the two treatment groups (Love et al., 2014). All differentially expressed genes underwent functional annotation using DAVID (Sherman et al., 2022; Huang et al., 2009). For overrepresentation analysis, the differentially expressed genes were first divided into two groups prior to analysis: Upregulated genes (log_2_-Fold Change > 0) and downregulated genes (log_2_-Fold Change < 0). The Gene Ontology (GO): Biological Processes terms with the clusterProfiler (v4.2.2) R package were used for analysis (Boyle et al., 2004; Wu et al., 2021), which used the hypergeometric distribution to determine whether any GO terms were overrepresented in each group than would be expected due to chance. Next, a transcriptome-wide enrichment analysis using the Hallmark gene sets from the Molecular Signatures Database was performed using the Gene Set Enrichment Analysis tool (Mootha et al., 2003; Liberzon et al., 2011; Korotkevich et al., 2021; Subramanian et al., 2005; Liberzon et al., 2015). The Hallmark gene sets represent specific biological states that are supported by substantial existing experimental evidence and have been generated through a combination of automated computational procedures and manual curation (Liberzon et al., 2015). The transcriptome-wide enrichment analysis used a ranked gene list of our entire dataset based on the log_2_-fold change values generated with DESeq2 but was undertaken independently of any significance testing from this analysis. The results from these analyses were visualised using ggplot2 (v3.4.2) (Wickham, 2016), EnhancedVolcano (v1.12.0) (Blighe et al., 2021), and PCAtools (2.6.0) (Blighe and Lun, 2021). To compare the transcriptomes of HCT-8 cells cultured within microfluidic device (infected with *C. hominis* vs. uninfected), initial sequencing data quality assessment, transcript abundance quantification and generation of gene level counts was performed as described above, with the exception that RUVseq was not used for normalisation and no batch effect correction was required. Differential expression analysis was performed using DESeq2 (v1.34.0) under default settings and at a false discovery rate (FDR) of 0.05 (Love et al., 2014). Data visualisation and functional annotation of differentially expressed genes was performed as described above. Overrepresentation analysis and gene set enrichment analysis was also undertaken as described above, with the exception that the Hallmark gene sets from the Molecular Signatures database was also used for overrepresentation analysis.

## 3 Results

The gut-on-chip utilised in this study employed pumpless and tubeless technology to enable application of a constant fluid shear stress of 0.02 dyn cm^-2^, using a hydrophilic spunlace thread to drive the fluid flow through the microchannel using controlled evaporation (Delon et al., 2020; Gunasekera et al., 2024). The HCT-8 monolayers were initially established in each microchannel without the thread to enable adherence to the Matrigel-coated glass surface. After fluid flow was initiated via insertion of the thread into the outlet, confluent monolayers were formed within three to five days of culture under the applied fluid shear stress. Scanning electron micrographs of the HCT-8 cell line cultured under fluid shear stress indicated that the HCT-8 monolayer grown on the Matrigel-coated surface of the microchannel exhibited enhanced three-dimensional topography and abundant microvilli-like structures (**Figure 1B**).

The transcriptomes of HCT-8 cells cultured within standard cell culture plates and microfluidic devices were characterised using a bulk-RNAseq approach. The data demonstrated clear differences between the two growth conditions. Principal component analysis of the gene-level count data showed distinct clustering of the two groups (**Figure 1C**). To estimate statistical significance, the results of the principal component analysis were compared against null distributions for statistics ψ (Vieira, 2012) and φ (Staelin and Gleason, 1975) (built using random permutations) (Camargo, 2022), which found that only PC1 accounted for a statistically significant proportion of the total variation (empirical ψ = 23.1370, maximum null ψ = 0.0031, minimum null ψ = 0.0003, *p*-value = < 0.05, empirical φ = 0.8782, maximum null φ = 0.0101, minimum null φ = 0.0034, *p*-value = < 0.05). The differential expression analysis of transcriptome-wide count data using DESeq2 revealed 219 differentially expressed genes between the HCT-8 cells cultured under fluid shear stress conditions compared to the HCT-8 cells cultured under static conditions. Of the genes that were identified as differentially expressed, 177 were upregulated and 42 were downregulated (**Figure 1D**).

Hypergeometric enrichment analysis of the 177 significantly upregulated genes indicated that 12 Gene Ontology (GO): Biological Processes terms (FDR ≤ 0.05), were overrepresented in this group (**Figure 1E**). No overrepresented GO terms were identified by analysis of the 42 downregulated genes. A transcriptome-wide gene set enrichment analysis, undertaken independently of statistical significance determined by differential gene expression analysis, indicated that nine Hallmarks were enriched in the ranked gene list (**Figure 1F**). Of these nine Hallmarks, oxidative phosphorylation had the highest normalised enrichment score, and mitotic spindle had the lowest.

**Figure 2:**
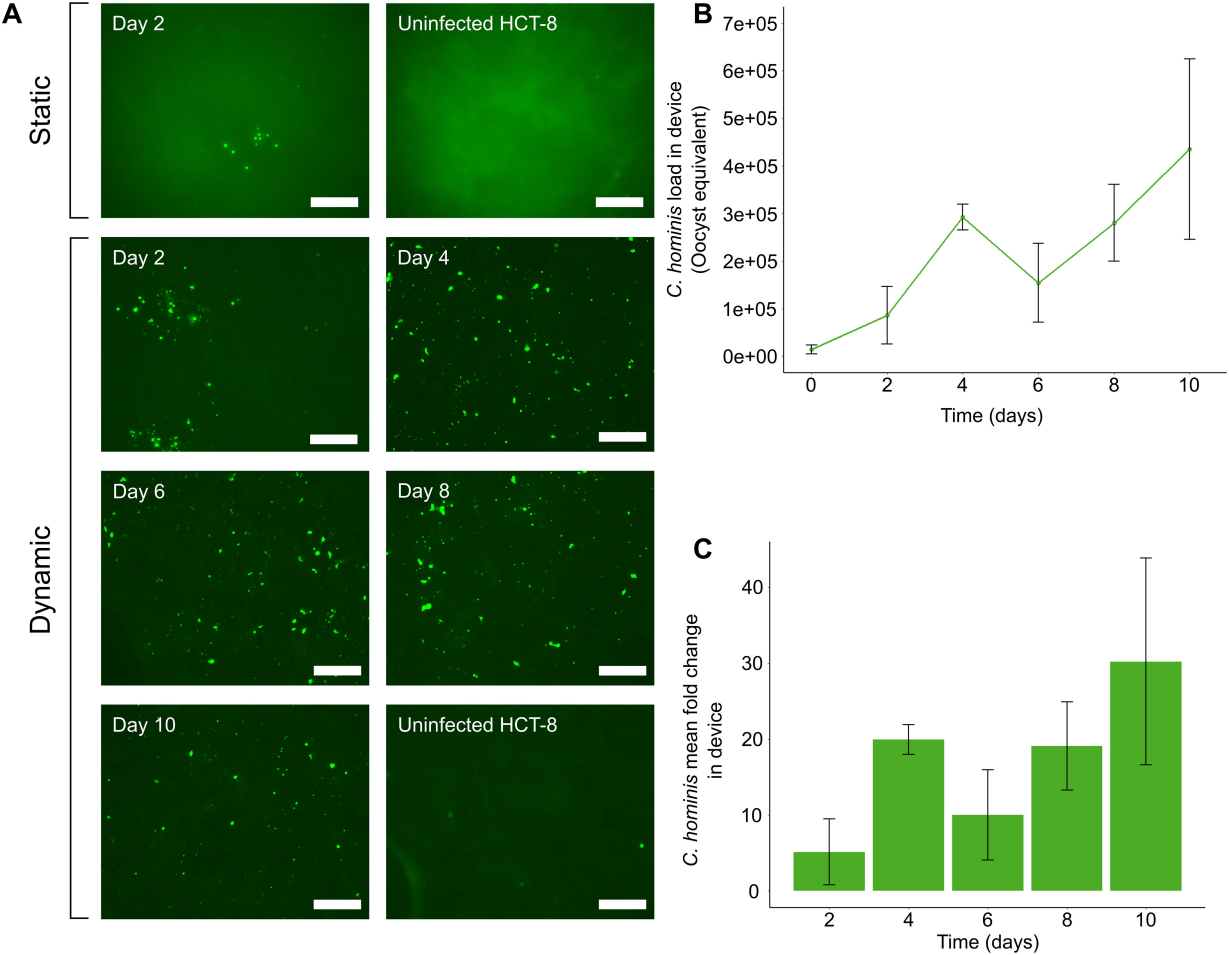
The microfluidic device supports *in vitro* development of *C*. *hominis* for up to 10 days. **(A)** Immunofluorescence data demonstrating foci of infection present at two days post-infection using the static system. Excystation pre-treated *C*. *hominis* oocysts were applied to HCT-8 monolayers grown in cell culture plates and microfluidic devices, then subjected to methanol fixation and immunostaining with Sporo-Glo at 48 h intervals. Post-processing of images was performed using Adobe Photoshop CS6 v13.0 with the following settings: shadow input 7, midtone input 1.8, highlight input 70. **(B)** qPCR data indicating expansion of *C*. *hominis* load over 10 days under a constant fluid shear stress of 0.02 dyn cm^2^. Excystation pre-treated oocysts were applied to HCT-8 cells cultured with fluid shear stress, and the infection medium, thread and monolayer were tested at 48 h intervals. The parasite load at each time-point was calculated from a standard curve. The data shown are the means and standard deviations from three microchannels per time-point. **(C)** Mean fold-change of *C*. *hominis* parasite load from initial inoculum when cultured under a constant fluid shear stress of 0.02 dyn cm^-2^. Error bars represent the standard deviation for each time-point. Scale bars = 100 µm.

Subtyping of all *C. hominis* oocyst isolates from human clinical cryptosporidiosis samples at the *gp60* locus revealed that all oocysts used in the experiments described herein were of subtype IdA15G1. After *C. hominis* infection was established in each microchannel of the gut-on-chip, and each plate well, immunofluorescence imaging was undertaken at each 48 h interval to determine the presence of actively dividing *Cryptosporidium* intracellular life cycle stages at each time-point. In the static system, foci of infection were present at Day 2 (**Figure 2A**), but clearly absent by Day 4 (data not shown). In contrast, in the gut-on-chip, the immunofluorescence data demonstrated that active *in vitro* development of *C. hominis* could be sustained for 10 days (**Figure 2A**).

Having established the possibility of extended culture of *C. hominis* within the gut-on-chip using the immunofluorescence assays, the total parasite load within the gut-on-chip was next measured at 48 h intervals for 10 days, using a *C. hominis*-specific qPCR assay. The qPCR assay demonstrated a substantial increase in parasite load within gut-on-chip devices over the entire duration of the experiment (**Figure 2B**), in agreement with the immunofluorescence data. The qPCR data from the gut-on-chip demonstrated a ~30-fold increase over a 10-day duration (**Figure 2C**). Sampling ceased after 10 days due to degradation of the HCT-8 cell monolayer over time.

**Figure 3:**
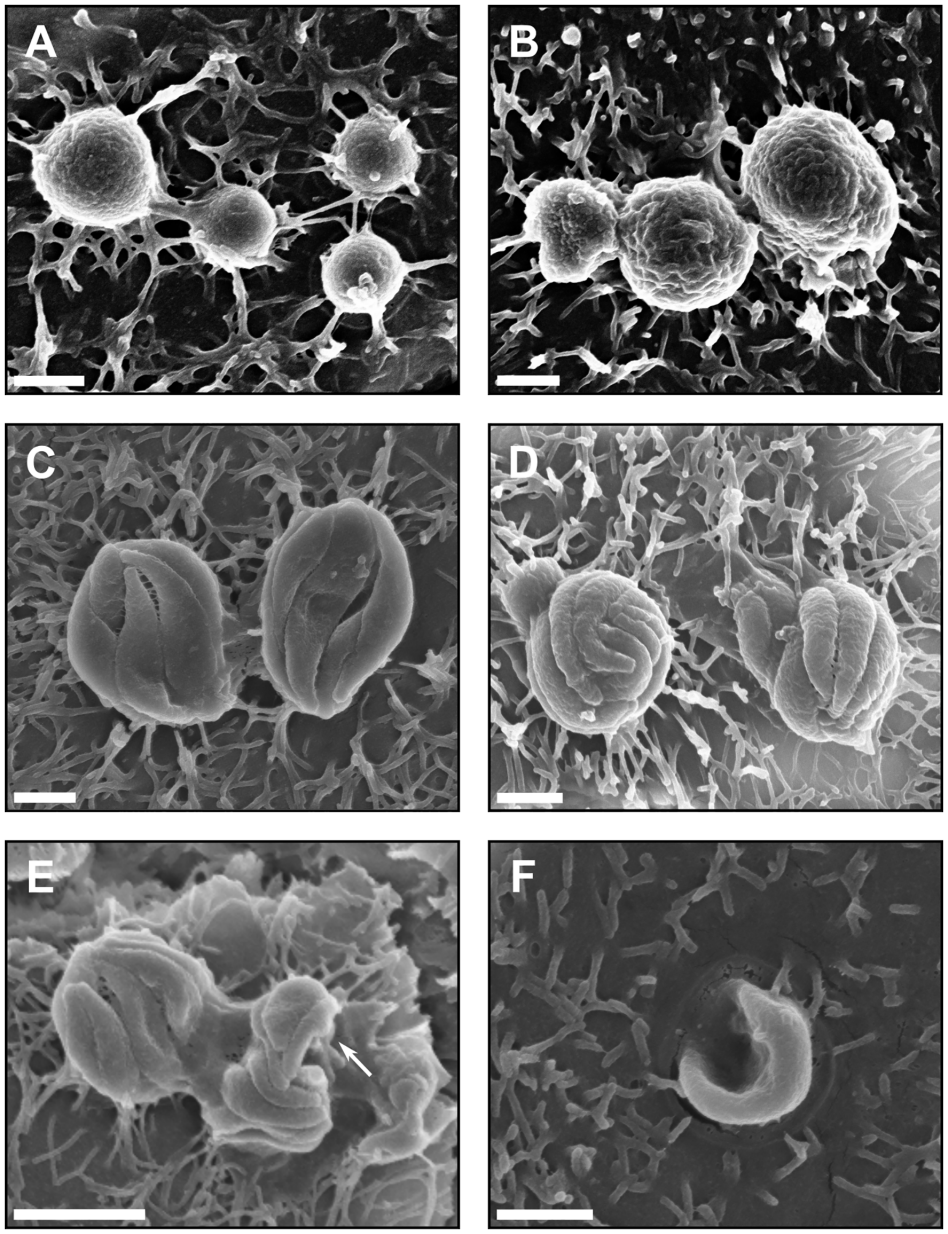
Asexual development of *C*. *hominis* occurs within the microfluidic device. **(A)** *C*. *hominis* trophozoites on the apical surface of the host cell monolayer measuring approximately 1 µm in diameter, **(B)** *C*. *hominis* trophozoites on the host cell surface, approximately 2 µm in diameter, **(C)** *C*. *hominis* meronts measuring approximately 3-4 µm in diameter with four internal merozoites visible, **(D)** *C*. *hominis* meronts measuring approximately 2-2.5 in diameter with eight internal merozoites visible, **(E)** *C*. *hominis* meronts with merozoites in egress (indicated by arrow), approximately 0.5 x 1 µm in size, **(F)** A free *C*. *hominis* merozoite invading the apical surface of the host cell, approximately 0.5 x 3 µm in size. Scale bars: **(A–D, F)** = 1 µm, **(E)** = 2 µm.

Next, a SEM approach was utilised to identify which *C. hominis* life cycle stages were present in the HCT-8 cell cultures under fluid shear stress conditions. These data indicated the presence of all common *C. hominis* life cycle stages at two days post-infection. The trophozoites were attached to the apical surface of the HCT-8 cell monolayer, observed to be spherical in shape, and ranged in size from 1-2 µm in diameter (**Figures 3A, B**). The meronts were comparable to trophozoites in cellular location, shape and size, but were distinct in appearance due to the presence of clearly visible internal merozoites (**Figures 3C–E**). The number of internal merozoites visible ranged between four and eight, and the observed size of each internal merozoite varied between 0.25-1 µm in width and 0.8-3 µm in length. Observations of merozoites in egress were rare but present (**Figure 3E**), and observations of free merozoites were uncommon but distinct in appearance due to the presence of pointed apical ends (**Figure 3F**).

**Figure 4:**
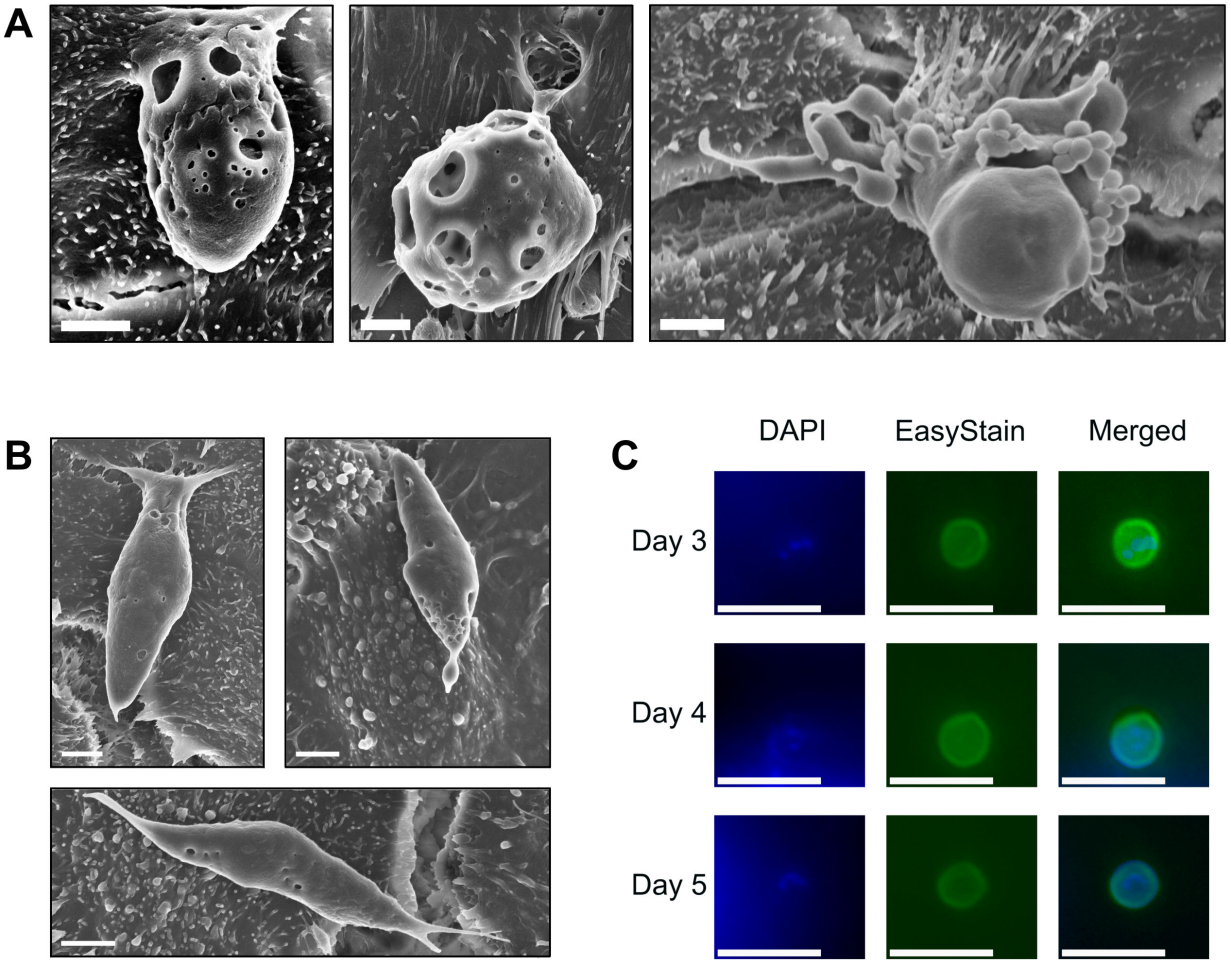
Sexual development occurs within the microfluidic device, but robust fertilisation remains absent. **(A)** *C*. *hominis* macrogamont attached to the host cell surface measuring approximately 4 x 6 µm in size (left) *C*. *hominis* macrogamont attached to the host cell surface measuring approximately 8 µm in diameter (middle), *C*. *hominis* microgamont attached to the host cell via a stalk with ~20 microgamete-like structures budding from the surface (right). Scale bars = 2 µm. **(B)** Possible *C*. *hominis* gamont-like life cycle stage approximately 12 µm x 4 µm in size (left and right), possible *C*. *hominis* gamont-like life cycle stage approximately 14 µm x 2.5 µm in size (bottom). Scale bars = 2 µm. **(C)** Contents of one microfluidic device at three-, four-, and five-days post-infection stained with DAPI and EasyStain, and merged channel image. Merged channel images underwent post-processing using Adobe Photoshop CS6 v13.0 using a green levels adjustment layer with the following settings: shadow input 7, midtone input 1.8, highlight input 70. Scale bars = 10 µm.

Scanning electron microscopy data indicated a large amount of diversity of asexual and sexual developmental stages by two days post-infection. Macrogamonts were frequently observed attached to the HCT-8 cell monolayer, measuring approximately 4-8 x 6-8 µm in size, and had numerous round-shaped perforations on the outer surface (**Figure 4A**). Observations of microgamonts were less frequent and were observed to be attached to the host cell monolayer via a stalk with up to 20 bullet-shaped microgamete-like structures budding from the surface (**Figure 4A**). Scanning electron microscopy also revealed the presence of much larger structures that were approximately 12-14 µm in length, 2.5-4 µm in width, with clear attachment points to the HCT-8 host cell monolayer visible (**Figure 4B**). Observations of the larger putative gamont-like life cycle stages were far more frequent than the sexual stages of development and most of the asexual developmental stages were present in all biological replicates, and completely absent in uninfected HCT-8 monolayers. Experiments designed to determine the presence of new oocysts produced within the microfluidic device were undertaken by purifying the sporozoites following excystation pre-treatment. Experiments were carried out over a one-week duration and yielded very low levels of oocysts produced *in vitro* at three-, four- and five- days post-infection (**Figure 4C**). An attempt to passage the culture at three days post-infection was unsuccessful with no foci of infection detected using Sporo-Glo (data not shown).

**Figure 5:**
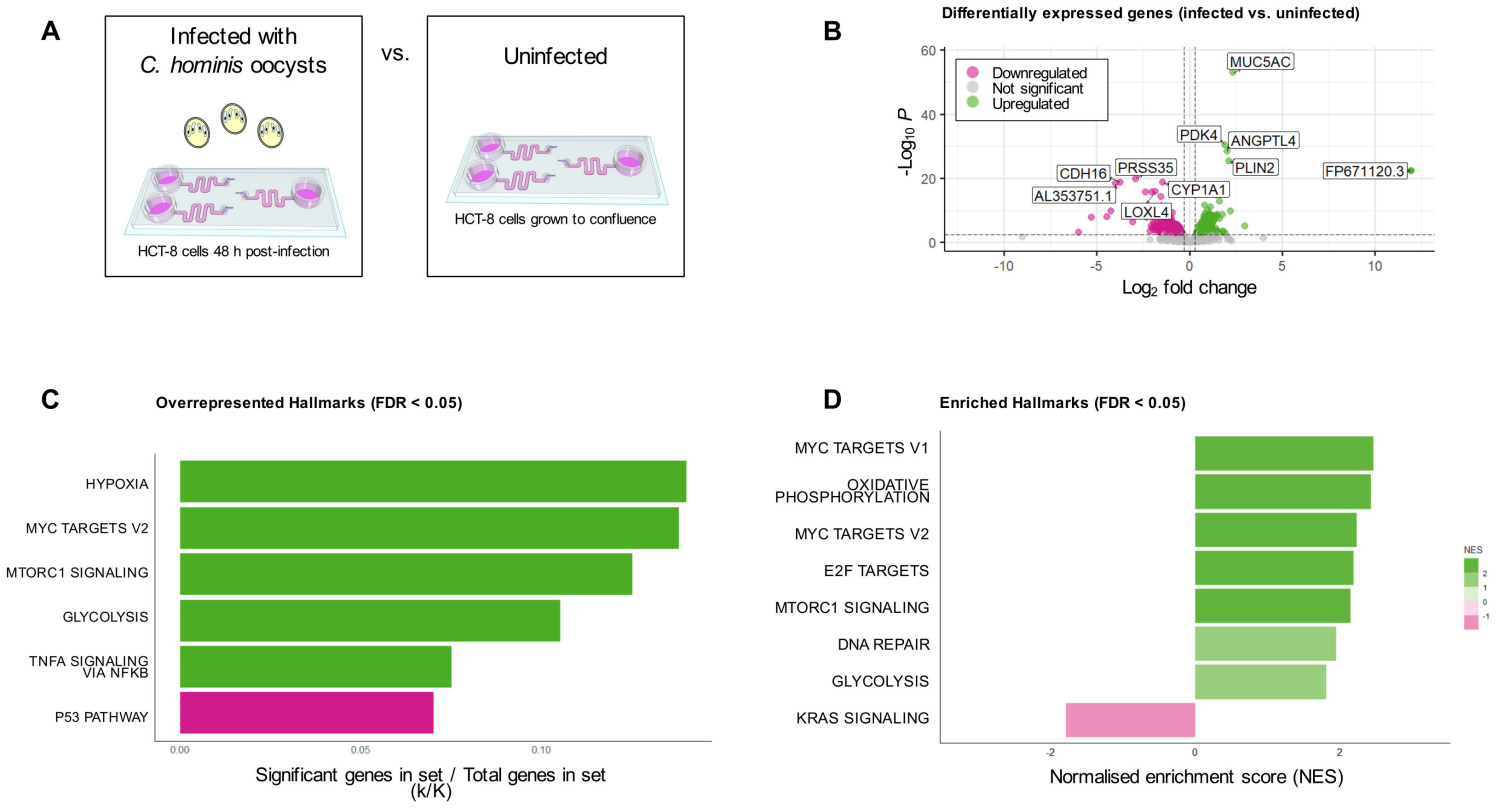
Analysis of HCT-8 cell transcriptome under fluid shear stress, with and without *C*. *hominis* infection. **(A)** Infographic of treatment groups for bulk transcriptomic analysis. **(B)** Differential expression analysis indicated that 791 genes were differentially expressed genes between the two groups (FDR < 0.05), with the five top up- and downregulated genes annotated on the plot, **(C)** Overrepresentation analysis of the 450 significantly upregulated genes demonstrated that five Hallmarks were overrepresented in this subset (shown in green), while overrepresentation analysis of the 341 downregulated genes revealed one overrepresented Hallmark in this subset (shown in pink), **(D)** Transcriptome-wide gene set enrichment analysis demonstrated the presence of eight enriched Hallmark gene sets (FDR < 0.01). Genes with a positive NES are shown in green, and those with a negative NES are shown in pink.

To investigate the host-parasite interaction occurring within the microfluidic gut-on-chip, a transcriptomics approach was utilised to compare the transcriptomes of the uninfected and *C. hominis*-infected HCT-8 cell line cultured within the microfluidic gut-on-chip (**Figure 5A**). Differential expression analysis detected the presence of 791 statistically significantly differentially expressed genes (FDR < 0.05), of which 450 were upregulated and 341 were downregulated (**Figure 5B**). Overrepresentation analysis of these 450 significantly upregulated genes using the Hallmark gene sets from the Molecular Signatures Database indicated that the hypoxia, Myc targets v2, mTORC1 signalling, glycolysis and TNF-α signalling via NFκβ Hallmarks were significantly overrepresented, and overrepresentation analysis of the 341 significantly downregulated genes revealed that the p53 pathway Hallmark was overrepresented (FDR < 0.05, **Figure 5C**). Transcriptome-wide gene set enrichment analysis using a ranked gene list based on log_2_ fold change against the Hallmark gene sets from the Molecular Signatures Database found that 15 Hallmarks were significantly enriched (FDR < 0.05, **Supplementary Figure 1**), with eight of these under an FDR of 0.01 (**Figure 5D**). There were four Hallmarks that were both overrepresented among the significantly differentially expressed genes and enriched based on gene set enrichment analysis of the entire transcriptome; these were Myc targets v2, glycolysis, mTORC1 signalling and hypoxia (**Supplementary Table 1**).

## 4 Discussion

Despite being the most common species of *Cryptosporidium* infecting humans in most parts of the world (Yang et al., 2021), there is remarkably little knowledge of the *in vitro* growth characteristics of *C. hominis*. There have been numerous advances in developing *in vitro* culturing platforms that support complete *C. parvum* development (Morada et al., 2016; DeCicco RePass et al., 2017; Baydoun et al., 2017; Heo et al., 2018; Wilke et al., 2019; Lamisere et al., 2022; Nikolaev et al., 2020), with substantially less culture systems demonstrating support of *C. hominis* (Hijjawi et al., 2010, 2001; Greigert et al., 2024). This study demonstrates that the extended *in vitro* development of *C. hominis* is possible for up to 10 days, using a simplified gut-on-chip where the integration of pumpless and tubeless microfluidics with conventional HCT-8 culture enabled continuous perfusion of cell culture medium through the microchannel, exposing the monolayers to a constant fluid shear stress of 0.02 dyn cm^-2^.

Through the fine control of mechanical and microfluidic forces within an *in vitro* environment, the application of fluid shear stress using microfluidics to model the gut milieu has been strongly associated with accelerated cell differentiation (Sontheimer-Phelps et al., 2019; Trietsch et al., 2017), enhanced villus formation (Kim and Ingber, 2013; Chi et al., 2015), heightened transcytotic activity (Delon et al., 2023), and successful co-culture of host commensal bacteria (Kim et al., 2012, 2016; Jalili-Firoozinezhad et al., 2019; Shah et al., 2016). As a result, there have been several advances in modelling the intestinal pathophysiology of many enteric pathogens using microfluidic culture systems (Tovaglieri et al., 2019; Chi et al., 2015; Grassart et al., 2019; Sunuwar et al., 2020; Villenave et al., 2017). Previous work demonstrated that *C. hominis* infects primary human duodenal epithelial cells more efficiently than HCT-8 cells (Hashim et al., 2004), illustrating the importance of prioritising physiological relevance for an enhanced *in vitro C. hominis* culture system. Additionally, microfluidics enabled long-term and complete *C. parvum* development using murine intestinal stem cell-derived cultures with constant perfusion of the cell culture medium (Nikolaev et al., 2020). The present study aimed to leverage the benefits of microfluidics, while ensuring the culture system remained accessible to most laboratories already using the HCT-8 infection model for *Cryptosporidium* culture.

In the present study, qPCR data demonstrated incremental rises in parasite load over time, and immunofluorescence assays demonstrated that actively dividing *Cryptosporidium* life cycle stages were consistently present for the 10-day duration of the experiment. Static comparison experiments described in the present study, indicated that *C. hominis* infection of the HCT-8 cell line under static conditions had terminated between Day 2 and Day 4 of infection; an observation which was in line with previous investigations of *Cryptosporidium* infection kinetics of the HCT-8 cell line (English et al., 2022; Tandel et al., 2019). In contrast, earlier research reported that *C. hominis* could be maintained in HCT-8 culture for up to 25 days using a combined approach of gamma irradiation of the cell monolayer prior to infection and passaging the cultures (Hijjawi et al., 2001). In agreement with the results described herein, most other studies observed limited *C. hominis* development using the HCT-8 cell line under static conditions for much shorter periods of time (Hashim et al., 2006, 2004; Stratakos et al., 2017; Chao et al., 2018). The axenic culture of *C. hominis* has also been reported, albeit with comparatively slower life cycle progression, and at best demonstrated a 5-6-fold increase in *C. hominis* parasite load over a nine-day period (Hijjawi et al., 2010). Additionally, a human ileal stem cell-derived air-liquid interface culture system also demonstrated sustained *C. hominis* development for up to nine days, with ~80-fold amplification occurring after three days of infection, which declined over the duration of the experiment (Greigert et al., 2024).

All common developmental stages of *C. hominis* were present by 48 h post-infection using the gut-on-chip, which were identified based on existing morphological information (Borowski et al., 2010). No quantitation of the proportion of each developmental stage was attempted using these data due to the non-random manner in which the images were acquired. Notably, the scanning electron micrographs of the macrogamont-like structures had distinct round-shaped perforations on the cell surface. It is currently unknown whether these perforations on the macrogamont surface are a biological feature or a product of sample processing. Given that these features were only present on the macrogamont-like structures, and absent in all other life cycle stages, the authors hypothesise that this is more likely to be a biological feature, but the significance of this observation is still yet to be elucidated.

The scanning electron microscopy data also suggested the presence of possible gamont-like structures as part of the *C. hominis* life cycle. In the absence of clear, definitive morphological features that characterise gamonts, the authors hypothesise that these structures may be gamonts given their unusually large size (12-14 µm in length) and similar elongated shape to previous reports (Hijjawi et al., 2002, 2004). Given that a *Cryptosporidium*-specific approach was not utilised (Edwards et al., 2012; Koh et al., 2014), and the lack of available morphological information on this rarely observed life cycle stage, the classification of these structures as gamont-like is presumptive. However, these possible gamont-like structures were present in all *C. hominis* cultures that were visualised using scanning electron microscopy over multiple biological replicates, and completely absent in uninfected HCT-8 monolayers, thus describing their presence in our system is warranted. These larger structures also coincide with larger surface areas of Sporo-Glo staining than what is typically observed in *C. parvum* immunofluorescence assays. Gamont-like developmental stages were first reported in *C. andersoni* and *C. parvum* in both HCT-8 cultures and in faecal matter from the *in vivo* host (Hijjawi et al., 2002), and later in axenic *C. parvum* cultures (Hijjawi et al., 2004; Aldeyarbi and Karanis, 2016) and cell free aquatic biofilm systems (Koh et al., 2014). Ultrastructural information of *C. parvum* gamonts has indicated that they may be double membrane-bound and multi-nucleated (Aldeyarbi and Karanis, 2016). The possible gamont-like structures observed in the present study were similar in shape and size to previous descriptions (Hijjawi et al., 2004, 2002), with an elongated shape and tapered anterior and posterior ends. In the present study, these larger structures were located epicellularly with clear attachment points to the host cell.

While a ~30-fold amplification was observed over a 10-day period, immunofluorescence assays indicated relatively low numbers of foci of infection, which may have been partially due to the effect of the fluid shear stress on parasite distribution across the monolayer, and exacerbated by a weaker fluorescent signal produced with Sporo-Glo staining of *C. hominis* compared to *C. parvum* (Ogbuigwe et al., 2023). An attempt to passage the cultures was unsuccessful, and the present study provided very limited evidence to support new oocyst formation *in vitro*. Given the reliance of the present experiments on *Cryptosporidium*-positive faecal samples, and the unusually low number of *C. hominis* cases over the last three years potentially due to the COVID-19 pandemic (Knox et al., 2021; Adamson et al., 2023), access to fresh *C. hominis* oocysts was limited. The suboptimal age of the *C. hominis* oocysts may have reduced their infectivity, leading to lower levels of sporozoite invasion, reduced life cycle progression and consequently lower numbers of oocysts arising from fertilisation occurring *in vitro*. Additionally, the numbers of oocysts obtained from *Cryptosporidium* cultures arising from purified sporozoites were likely too low to accurately assess their *in vitro* infectivity. Future experiments should repeat such attempts with freshly harvested oocysts.

Transcriptomic analysis of the HCT-8 cell line cultured within the gut-on-chip demonstrated a metabolic shift towards oxidative phosphorylation. Elevated mitochondrial ATP synthesis and mitochondrial biogenesis in response to shear stress has been previously well-documented in human endothelial cells (Yamamoto et al., 2011, 2018, 2020; Li et al., 2009; Kim et al., 2015; Wu et al., 2018), and many of these reports documented no impact on cytosolic glycolysis (Yamamoto et al., 2018, 2020; Shen et al., 2018). Previous work investigating the impacts of fluid shear stress on Caco-2 cell monolayers was indicative of a positive relationship between mitochondrial activity and fluid shear stress (Delon et al., 2019, 2020). The changes to the cell cytoskeleton elicited by fluid shear stress are highly energetically expensive (Dawson et al., 2023), and the upregulated oxidative phosphorylation and fatty acid metabolism observed in HCT-8 cells within the gut-on-chip in the present study may be a necessary metabolic adaptation of the cell to maintain enhanced apical surface structure. Upregulated fatty acid metabolism in response to fluid shear stress has been previously reported in kidney epithelial cells, and these metabolic changes were identified to be indispensable in supporting shear stress-induced actin cytoskeleton remodelling (Miceli et al., 2020). The observed metabolic changes in the host cell may indirectly form part of the biological basis of enhanced *Cryptosporidium* development using this system by supporting enhanced apical surface structure of the host cell.

Clear transcriptomic differences were also observed in *C. hominis-*infected HCT-8 cells cultured in the microfluidic device when compared to the uninfected HCT-8 cell line cultured under the same conditions. There are limited existing investigations of the HCT-8 transcriptome in response to *C. hominis* infection specifically. Other RNA-seq and microarray studies observing the HCT-8 cell response to *C. parvum* infection at various stages of infection demonstrated a much lower number of differentially expressed genes between the infected and uninfected HCT-8 cell (Jiménez-Meléndez et al., 2023; Sun et al., 2022; Yang et al., 2009). Given that 791 significantly differentially expressed genes were identified in the present study, this observation suggests either that *C. hominis* may recruit a larger host response than *C. parvum*, or that the application of fluid shear stress amplifies the differences between the infected and uninfected HCT-8 cell transcriptome. In contrast to this observation, an RNA-seq study using a human ileal stem cell-based culture system for *Cryptosporidium* found that *C. parvum* recruited a larger host response than *C. hominis*, though this was partially attributed to higher viral load in the *C. parvum* isolate (Greigert et al., 2024).

The most statistically significantly upregulated gene in HCT-8 cells infected with *C. hominis* (IdA15G1) was the mucin encoding *MUC5AC* gene. While increased *MUC5AC* expression has not been linked to the host response to *Cryptosporidium* previously, it has been identified as a critical component of the host response to *Trichuris* infection for parasite clearance (Hasnain et al., 2011, 2012, 2010). There is also evidence supporting upregulated host *MUC5AC* expression in response to *Giardia duodenalis* infection (Amat et al., 2017). Further functional assays should be undertaken in the future to elucidate the role of mucin production in the host response to *Cryptosporidium* infection. Additionally, our data indicated that the *LOXL4* gene was significantly downregulated in response to *C. hominis* infection. A previous study indicated that host *LOXL4* expression is suppressed in response to *C. parvum* infection as a result of importation of the parasite Cdg7_FLc_1000 RNA into the host cell nucleus (Ming et al., 2018).

Both overrepresentation analysis (limited to statistically significantly differentially expressed genes) and transcriptome-wide gene set enrichment analysis were undertaken using the Hallmark gene sets in the Molecular Signatures Database (Liberzon et al., 2011, 2015). The present study found that the hypoxia, mTORC1 signalling, TNF-α signalling via NFκβ, and glycolysis Hallmarks were overrepresented among the 450 significantly upregulated genes in *C. hominis*-infected HCT-8 cells, and the p53 pathway Hallmark was overrepresented in the 341 significantly downregulated genes. Transcriptome-wide gene set enrichment analysis found that the oxidative phosphorylation, E2F targets, Myc targets, DNA repair, KRAS signalling Hallmarks, in addition to mTORC1 signalling and glycolysis, were significantly enriched in *C. hominis*-infected HCT-8 cells. There is some evidence linking *C. parvum* and *C. muris* infection with the induction of the response to hypoxia, though the significance of these observations remains unexplored (DeMichele et al., 2023). A recent RNA-seq study investigating *C. parvum* infection in neonatal bovines also found upregulated NFκβ activity in response to infection (Veshkini et al., 2024). Additionally, the pro-inflammatory cytokine TNF-α has been previously linked to the host response to *Cryptosporidium* (Zhao et al., 2016; Seydel et al., 1998), but whether it is essential for parasite clearance (Lean et al., 2006; Lacroix et al., 2001), and its association with clinical symptoms (Robinson et al., 2001), is unclear. A previous study only found the interferon-α response Hallmark to be significantly enriched in *C. parvum* cultures under fluid shear stress (Nikolaev et al., 2020), which was supported by a recent study demonstrating upregulation of both type I and type II interferon signalling in response to *Cryptosporidium* infection (Greigert et al., 2024). Surprisingly, neither overrepresentation analysis nor gene set enrichment analysis recovered the same finding in the *C. hominis*-infected HCT-8 cultures in the present study. Although the majority of our knowledge of *Cryptosporidium in vitro* growth characteristics has been based on *C. parvum*, there are some known differences in how *C. hominis* development progresses *in vitro*. *C. hominis* has been documented to infect the HCT-8 cell line with less efficiency and less uniformity than *C. parvum* (Hashim et al., 2004). A human stem cell-based system that was able to support both *C. hominis* and *C. parvum* also demonstrated that *C. hominis* fold growth over time was lower when compared to *C. parvum* (Greigert et al., 2024). In contrast, *C. hominis* has also been reported to develop at a faster rate than *C. parvum* within the HCT-8 model (Hijjawi et al., 2001). The ability for long-term culture of *C. hominis* will facilitate further experiments of the *in vitro* growth characteristics of this species as well as broader investigation of the biology and life cycle of *Cryptosporidium*.

This study reports the use of a bioengineered gut-on-chip for extended *in vitro* development of *C. hominis* for up to 10 days. Analysis of the host HCT-8 cell line cultured within the gut-on-chip indicated a metabolic shift towards oxidative phosphorylation under microfluidic conditions. Scanning electron micrographs of infected HCT-8 monolayers demonstrated the presence of trophozoites, meronts, merozoites, macrogamonts and microgamonts. SEM also suggested the presence of larger gamont-like putative life cycle stages. Infection trials utilising purified sporozoites yielded very low levels of oocysts produced *in vitro*, but future studies will aim to optimise the *in vitro* microenvironment to enable robust oocyst formation. This study indicates the importance of fluid shear stress in developing *in vitro* models for *Cryptosporidium* infection that more accurately recapitulate the complex *in vivo* environment. We anticipate that the use of the gut-on-chip for *Cryptosporidium* infection will foster future studies of the biology and life cycle progression of *C. hominis*.

## Data Availability

Sequencing data were uploaded to the NCBI SRA database (BioProject accession number PRJNA1051824). All other raw data supporting the conclusions of this article will be made available by the authors, without undue reservation.
