# Supplementary material for: Microphysiological gut-on-chip enables extended in vitro development of Cryptosporidium hominis
Source: Front Cell Infect Microbiol. 2025 Apr 24;15:1564806. doi: 10.3389/fcimb.2025.1564806 (PMC12058726; doi:10.3389/fcimb.2025.1564806)
Supplement: Supplementary file 1 [file SupplementaryFile1.docx]

Supplementary Material


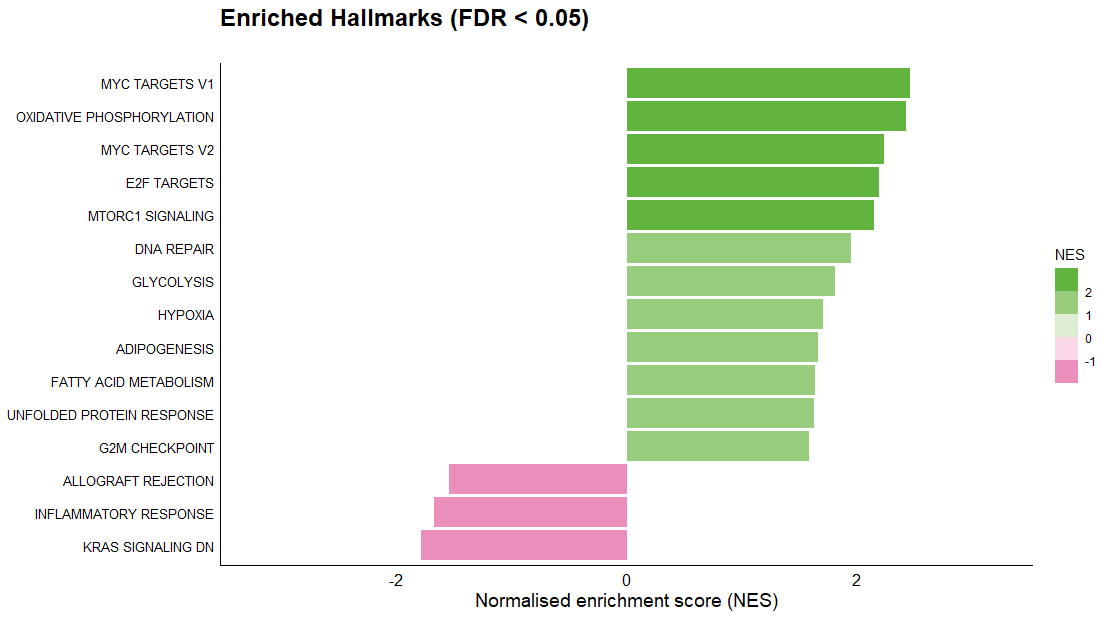


**Supplementary Figure 1:** Results of the gene set enrichment analysis using Hallmark gene sets (FDR ≤ 0.05)
