# Supplementary material for: Microphysiological gut-on-chip enables extended in vitro development of Cryptosporidium hominis
Source: Front Cell Infect Microbiol. 2025 Apr 24;15:1564806. doi: 10.3389/fcimb.2025.1564806 (PMC12058726; doi:10.3389/fcimb.2025.1564806)
Supplement: Supplementary file 2 [file Table1.docx]

**Supplementary Table 1:** **List of negative control genes used for normalisation with RUVseq**

| **Gene symbol** | **Gene name** |
| --- | --- |
| ACTB | Actin beta |
| B2M | Beta-2-microglobulin |
| BLM | BLM RecQ like helicase |
| GAPDH | Glyceraldehyde-3-phosphate dehydrogenase |
| PPIA | Peptidylprolyl isomerase A |
| RPL4 | Ribosomal protein L4 |
| YWHAZ | Tyrosine 3-monooxygenase/tryptophan 5-monooxygenase activation protein zeta |
| UBC | Ubiquitin C |
| RPL29 | Ribosomal protein L29 |
| RPLP0 | Ribosomal protein lateral stalk subunit P0 |
| PGK1 | Phosphoglycerate kinase 1 |
| HMBS | Hydroxymethylbilane synthase |
| HPRT1 | Hypoxanthine phosphoribosyltransferase 1 |
| SDHA | Succinate dehydrogenase complex flavoprotein subunit A |
| TBP | TATA-box binding protein |
| ALAS1 | 5'-aminolevulinate synthase 1 |
| PUM1 | Pumilio RNA binding family member 1 |
| GUSB | Glucuronidase beta |
| TFRC | Transferrin receptor |
| MALAT1 | Metastasis associated lung adenocarcinoma transcript 1 |
