# Supplementary material for: Microphysiological gut-on-chip enables extended in vitro development of Cryptosporidium hominis
Source: Front Cell Infect Microbiol. 2025 Apr 24;15:1564806. doi: 10.3389/fcimb.2025.1564806 (PMC12058726; doi:10.3389/fcimb.2025.1564806)
Supplement: Supplementary file 3 [file Table2.docx]

**Supplementary Table 2: Function of the top five up- and downregulated genes in HCT-8 cells under fluid shear stress.**

| Gene symbol | Gene full name | UniProtKB key words (biological process or disease) | Log_2_ FC | FDR |
| --- | --- | --- | --- | --- |
| SH3BGRL3 | SH3 domain binding glutamate rich protein like 3 | - | 6.60 | 5.59E-14 |
| FOS | FOS proto-oncogene, AP-1 transcription factor subunit | Proto-oncogene (KW-0656) | 5.82 | 7.23E-13 |
| SEC61G | SEC61 translocon subunit gamma | Protein transport (KW-0653), Translocation (KW-0811) | 4.36 | 4.02E-07 |
| CLIC3 | Chloride intracellular channel 3 | Ion transport (KW-0406) | 8.76 | 4.31E-07 |
| LCN2 | Lipocalin 2 | Apoptosis (KW-0053), Innate immunity (KW-0399), Ion transport (KW-0406) | 4.92 | 4.92E-07 |
| AC145207.3 | AC145207.3 | - | -19.37 | 1.24E-15 |
| ZBED6 | Zinc finger BED-type containing 6 | Transcription (KW-0804), Transcription regulation (KW-0805) | -6.31 | 2.66E-06 |
| ORAOV1 | LTO1 maturation factor of ABCE1 | - | -5.47 | 2.66E-06 |
| MRNIP | MRN complex interacting protein | DNA damage (KW-0227), DNA repair (KW-0234) | -8.36 | 2.55E-05 |
